# Integrating the Ergonomics Techniques with Multi Criteria Decision Making as a New Approach for Risk Management: An Assessment of Repetitive Tasks-Entropy Case Study

**Published:** 2016-06-12

**Authors:** Mohammad Khandan, Majid Nili, Alireza Koohpaei, Saeedeh Mosaferchi

**Affiliations:** ^a^ Department of Ergonomics, Faculty of Health, Qom University of Medical Sciences, Qom, Iran; ^b^ Department of Industrial Management, Faculty of Management, Qom university, Qom, Iran; ^c^ Department of Occupational Health & Work Health Research Center, Faculty of Health, Qom University of Medical Sciences, Qom, Iran; ^d^ Department of Ergonomics, Faculty of Health, Tehran University of Medical Sciences, Tehran, Iran.

**Keywords:** Multiple Criteria Decision Making, Entropy, Occupational ergonomics, Assessment of Repetitive Tasks Method

## Abstract

**Background:** Nowadays, the health work decision makers need to analyze a huge amount of data and consider many conflicting evaluation criteria and sub-criteria. Therefore, an ergonomic evaluation in the work environment in order to the control occupational disorders is considered as the Multi Criteria Decision Making (MCDM) problem. In this study, the ergonomic risks factors, which may influence health, were evaluated in a manufacturing company in 2014. Then entropy method was applied to prioritize the different risk factors.

**Methods:** This study was done with a descriptive-analytical approach and 13 tasks were included from total number of employees who were working in the seven halls of an ark opal manufacturing (240). Required information was gathered by the demographic questionnaire and Assessment of Repetitive Tasks (ART) method for repetitive task assessment. In addition, entropy was used to prioritize the risk factors based on the ergonomic control needs.

**Results:** The total exposure score based on the ART method calculated was equal to 30.07±12.43. Data analysis illustrated that 179 cases (74.6% of tasks) were in the high level of risk area and 13.8% were in the medium level of risk. ART- entropy results revealed that based on the weighted factors, higher value belongs to grip factor and the lowest value was related to neck and hand posture and duration.

**Conclusions:** Based on the limited financial resources, it seems that MCDM in many challenging situations such as control procedures and priority approaches could be used successfully. Other MCDM methods for evaluating and prioritizing the ergonomic problems are recommended.

## Introduction

High level of prevalence of disorders in musculoskeletal system in various organizations is a well-known issue and their negative consequences such as increase in costs and individual as well as organizational problems are proved by experts^1-4^. Different factors cause musculoskeletal disorders including job-related factors like unacceptable ergonomic conditions of workplace, manual tasks, heavy load handling, and repetitive tasks^5,6^.

Musculoskeletal disorders are multi-factor problem so severity of injury would be higher if different factors are present at the same time^6^. Therefore, it is important to identify and analyze a wider range of factors in order to prevent and to take a better control over musculoskeletal disorders especially at workplaces. There are different methods to assess repetitive tasks especially with a focus on upper limbs, for example OCRA, SI and CTD Index^7^.

One of the novel methods in this field is Assessment of Repetitive Tasks (ART). Compared with other methods, this tool assesses more risk factors of musculoskeletal problems. Despite the fact that higher numbers of assessed factors could help ergonomists to control and limit them, it would cause difficulties in analysis and decision-making process. In other words, usual methods in ergonomics such as MAC, RULA, LUBA and REBA are useful for risks recognition in companies^8^. However, any techniques have many criteria; for example, ART method has 12 criteria for assessment. Which of these factors really are more important than others are in an evaluated company? ART and other usual techniques cannot answer this question.

If systematically plan is considered, where to start and what factors? Decision-making models such as entropy help us to answer these questions. Entropy tells us which factors have higher effects on reducing the risk. This process continues until the end. Many control measures are stopped in health, safety and ergonomic (HSE) practices due to lack of budget especially in developing countries. Decision-making models take to stop this process and this is very important and attractive for the field of HSE. It seems that using decision-making models like Multi Criteria Decision Making (MCDM) can solve the problem. In a majority of MCDM issues we need to know the relative importance of criteria, in a way that their summation be one (normalized); relative importance depicts priority of each criterion among a list of criteria to decision-making^9^. There are sorts of methods such as Analytic Hierarchy Process (AHP) and entropy. One of the main assumptions is the validation of weights of studied criteria. Totally, methods used to weigh criteria are subjective or objective. Change in weights would affect the function of MCDM methods in finding the best alternative. In situations like this, it is critical to have a scientific criterion that is able to evaluate validity of weights and acceptability of results. Entropy has been used to weigh factors in different studies in various fields like manufacturing companies^10-12^, healthcare centers^13^ and mine^14,15^ and was approved.

The objective of this study was to evaluate ergonomics risk factors assessed by ART tool using entropy method in a manufacturing company in the central province of Iran, 2014.

## Methods

In this cross-sectional study, 240 subjects working in seven production saloons of an Arc Opal dishes manufacturing company (Mahfam Jam) located in the central part of Iran in 2014. Participants totally performed 13 tasks. All tasks were defined using formal documents in the company and their conditions were analyzed. Exclusion criteria illustrated by interview and self-reporting were problems in joints like arthritis, disc herniation, and fracture in spine or any other disorders in musculoskeletal system and pain in any part of the body. A researcher-developed questionnaire containing questions around age, sex, work experience, and ergonomics and/or work-related trainings was used to gather demographic data. In addition, at the first step an ergonomic risk factor evaluation was performed by assessment of repetitive tasks of the upper limbs (ART) method. Then with aided MCDM principles (Entropy), all ART factors (12 criteria) were analyzed and a priority list was created finally.

Statistical analysis was conducted using SPSS V.20 (Chicago, IL, USA).

ART method was developed by Health and Safety Laboratory (HSL) in collaboration with Health and Safety Executive (HSE) in 2007. It is an acceptable technique to survey upper limbs in repetitive activities^16^. Its efficiency and utilization has been approved by researchers^16^. It contains four main steps^17^ as frequency and repetition of movements, force, awkward postures and additional factors. They are evaluated based on both qualitative and quantitative criteria and sorted into three groups of Green color or low, medium or Amber color and Red color or high level of risk in qualitative assessment. In our quantitative analysis, each one had a specific score and the final score was calculated from zero to 72; 0-11 was low risk, 12-21 medium and more than 22 high risk^17^. Additional factors included five sub-factors: breaks: the maximum amount of time that individuals perform the repetitive task without a break, work pace: difficulties that workers might have been keeping up with the work, other factors such as inadequate lighting levels and the use of hand as a tool (e.g. hammer), duration of task by a worker in a typical day or shift, and psychosocial factors like little control over how the work is done, and monotonous work.

### Entropy Method

Entropy is a major conception in physics, social science, and information theory, which shows the amount of uncertainty (distribution of studied criteria or risk factors in the studied saloons of the company) in an expected informational content of a message. In other words, entropy in information theory is a criterion for uncertainty that was explained by a discontinuous probability distribution (p_i_). If distribution is wider, the uncertainty would be more than the sharper ones^10^. This uncertainty is given by eq. 1.

(Eq. 1) $$\text{E}\approx {\text{S\{p}}_{\text{1}}{\text{,p}}_{\text{2}}\text{,}....{\text{,p}}_{n}\}=-K\sum_{i=1}^{n}\left[p_{i}.Lnp_{i}\right]$$

Where K is a positive constant variable in order to supply 0 ≤ E ≤ 1. Zero shows lower degree of distribution. Since this is only an expression for entropy as found in statistical mechanics, it is known as entropy of the probability distribution p_i_^18^.

E was calculated from the probability distribution p_i_ by statistical mechanism and it was the maximum value if all of pis (pi=1n) are same. Therefore, eq. 2 will be obtained.

(Eq. 2) $$-K\sum_{i=1}^{n}\left[p_{i}.Lnp_{i}\right]=-K\{\frac{1}{n}Ln\frac{1}{n}+\frac{1}{n}Ln\frac{1}{n}+...+\frac{1}{n}Ln\frac{1}{n}\}=-K\{(Ln\frac{1}{n})(\frac{n}{n})\}=-K\;Ln\frac{1}{n}$$

A decision-making matrix of a MADM model contains data for which entropy can be used as a criterion to evaluate (Table 1).

**Table 1:** A decision-making matrix of a MADM model

	X_1_	X_2_	….	X_n_
A_1_	r_11_	r_12_	….	r_1n_
A_2_	r_21_	r_22_	….	r_2n_
⋮	⋮	⋮		⋮
A_m_	r_m1_	r_m2_	….	r_mn_

X: Criteria (there are 12 examined criteria in this study) A: Alternatives (there are 7 studied saloons as alternatives in this study)

The available data in the decision-making matrix will be normalized by eq. 3.

(Eq. 3) $$P=\frac{r_{ij}}{\sum_{i=1}^{m}r_{ij}};\forall i,j$$

In this study, p_ij_ is normalized score of each criterion (factors of ART method). In other words, it is the ratio of score of criterion i to summation of the criterion’s score in different saloons.

And for E_j_ from p_ij_ set in lieu of every specification we will have eq. 4.

(Eq. 4) $$e=-K\sum_{i=1}^{m}\left[p_{ij}.Lnp_{ij}\right];\forall j$$

Here, m equals seven (number of studied saloons). Therefore, K is a constant of 0.514.

Now uncertainty or deviation degree (d_i_) from obtained data in lieu of the j^th^ specification is so eq. 5. It depicts unbalance distribution of criteria. The higher d_j_, the further distribution of criterion j in the company.

(Eq. 5) $$d_{j}=1-E_{j};\forall j$$

Finally, regarding weights (w_j_) of existed specification we will have eq. 6.

(Eq. 6) $$w_{j}=\frac{d_{j}}{\sum_{j=1}^{n}d_{j}};\forall j$$

w demonstrates the importance of the criterion based on analyzed data from all saloons. Altogether, entropy helps to refine data and do not use raw scores to make decision and pay to corrective actions. Applying entropy is something like a catalyzer to modify information by comparing states of criteria in all parts of company and find about their distribution (in how many saloon, and in what level of risky state each factor has been found). It would help to have more effective actions to make priority to control risk factor all around the studied sections of the company.

## Results

Total number of studied employees was 240 for operational workers in seven production saloons. 51.6% of participants were female. The mean±SD of ages was 28.02±5.53 yr in the range of 18-57 yr. Mean ±SD of work experience was 4.54±3.72 yr, in addition to 0.64±0.71 for training courses on ergonomics. Description of educational levels and workers in different halls are presented in Table 2.

**Table 2:** Data on studied saloons and workers’ education level (n=240)

**Variable**s	**Frequency**	**Percent**
Hall		
Pars pack	47	19.6
Pars Naghsh	26	10.8
Packaging	3	1.2
Leher	28	11.7
Tempering	19	7.9
Gradation	33	13.8
Decoration	84	35.0
Education level		
Up to diploma	58	24.2
Diploma	137	57.1
Associate's degree	21	8.7
Bachelor and higher	24	10.0

In addition, ART scores were from six to 39 and the mean was 30.07 with 12.43 as SD. In addition, monotonous work, high levels of attention and concentration, frequent tight deadlines, and incentives to skip breaks or finish early were most frequent psychosocial factors.

Entropy was used to define the importance of each factor assessed in ART. Twelve factors (in ART method, n=12) in seven production saloons (m=7) were assessed. Hence, K was calculates as 0.514. Scores of each factor in each saloon were measured in order to conduct entropy (Table 3).

**Table 3:** Decision-making matrix based on gathered data using ART method

Criteria Alternatives	A_1_	A_2_	B	C_1_	C_2_	C_3_	C_4_	C_5_	D_1_	D_2_	D_3_	D_4_
Pars pack	6	6	5	2	2	0	2	0	8	2	2	1
Pars Naghsh	0	3	0	1	1	0	1	0	4	1	1	0.5
Packaging	6	6	8	2	1	2	2	1	8	2	1	1
Leher	3	6	0	2	2	4	2	2	0	1	0	1
Tempering	6	6	5	2	2	4	2	0	0	1	2	1
Gradation	3	3	0	2	0	0	2	0	0	1	2	1
Decoration	6	6	8	2	0	2	2	1	8	2	2	1

A_1_:arm movements, A_2_:repetition, B:force, C_1_:neck/head posture, C_2_:back posture, C_3_:arm posture, C_4_: wrist posture, C_5_:hand/finger grip, D_1_:breaks, D_2_:work pace, D_3_:other factors such as vibration, etc. D_4_: duration.

After calculating weights of factors, the highest one was 0.249 belonged to the hand/finger grip. On the other hand, the lowest weight was 0.006 related to neck head posture, wrist posture and duration. The Table 4 shows weight and importance of each factor.

**Table 4:** Weights and importance of ART factors based on Entropy

**Entropy items**	**Criteria**											
	A1	A2	B	C1	C2	C3	C4	C5	D1	D2	D3	D4
E_j_	0.989	0.902	0.970	0.705	0.549	0.989	0.694	0.809	0.989	0.709	0.980	0.902
d_j_	0.011	0.098	0.030	0.295	0.451	0.011	0.306	0.191	0.011	0.291	0.020	0.098
W_j_	0.054	0.011	0.160	0.006	0.106	0.169	0.006	0.249	0.163	0.017	0.054	0.006
Importance/ priority	6	8	4	9	5	2	9	1	3	7	6	9

Based on the entropy’s results, the criterion C5 (hand/finger grip) had the highest importance distributed in studied production halls. Therefore, if this factor is at the top of agenda the result of correction will be the best compared with other 11 criteria.

## Discussion

Based on entropy analysis results, whatever the significance of studied factor has been reduced, the effect of the relevant factors in deciding the appropriate option, has been reduced too. The working unit selection to implement corrective actions is an example^10^. This means that the impact factor for all options (working units) is almost identical. Thus, factors such as grip, arm posture and rest during shifts should be important in decision-making because it had the greatest weight. On the other hand, wrist posture, head / neck postures and working time (8 hours’ work shift) had the lowest weight and importance. Accordingly, these factors had less impact on decision-making process.

The weights obtained by entropy method for measured parameters in the art frame showed that waist posture had gained considerable weight (0.106) as fifth line in terms of importance. As a result, waist posture increases the risk of musculoskeletal disorders similar to another study^10^. Based on mentioned research (QEC data), 53% of subjects in waist, 58% in arm and shoulder, 79% in neck and 81% in wrist were placed in high-level risk area. Table 4 shows the similarity between our study situation for arm and waist (second and fifth line respectively) with that of Sarsangi et al.’s research^19^but results for neck and wrist were different because of the differences between ART and QEC.

Wrist posture gained a low weight contrary to a previous study^19^. Tint et al.^20^ have evaluated risk factors of Carpal Tunnel Syndrome (CTD) in repetitive tasks on the computer users. They applied ART method and showed that in uniform and repetitive activities, the probability to cause problems such as musculoskeletal disorders was high. This difference may be considered due to differences in the nature of work and negative postures while using the keyboard. In addition, the grip situation and postures in the present study had the highest weight^20^. This result was similar to that of Abbaszadeh et al.^21^. In addition, similar research regarding rest time with high weight can be allocated (third line).

## Conclusions

The use of entropy in multiple factor situations can help occupational specialists in enhancing the ergonomic risk control as well as prevention of incidents and prevalence of problems and subsequent consequences. According to the results of a combination of both art and entropy patterns and on the other hand, taking into account the demographic profile of employees surveyed (The majority of subjects were women with little work experience and limited training courses), the need to focus more on engineering and administrative controls, especially in equipment, training and reorganization of work, such as work-rest rhythm was detected. Further analysis about psychosocial factors in the company is recommended.

Since the financial resources in organizations (especially in health, safety and ergonomic sectors) are very limited hence, we need a priority list for decision making about our challenges in HSE. It seems that MCDM techniques such as entropy can help to resolve the health, safety and ergonomic challenges in an effective and productive manner.

## Acknowledgments

Researchers have considered it necessary to appreciate all participants in the project and Qom University of Medical Sciences for their financial support.

## Conflict of interest statement

Authors declare that have no conflict of interest.

## Highlights

- Based on the ART method, 74.6% of the studied tasks were high risk.
- MCDM techniques can help to resolve HSE problems.
- The entropy in multiple factor situations can help to enhance controlling ergonomic risk factors.
